# Capturing relevant extracellular matrices for investigating cell migration

**DOI:** 10.12688/f1000research.6623.1

**Published:** 2015-12-07

**Authors:** Patricia Keely, Amrinder Nain

**Affiliations:** 1 Department of Cell and Regenerative Biology, UW Carbone Cancer Center, UW School of Medicine and Public Health, University of Wisconsin-Madison, Madison, WI, USA; 2 2Department of Mechanical Engineering, Virginia Tech, Blacksburg, VA, USA

**Keywords:** cell migration, extracellular matrix, ECM, stromal microenvironment, Matrix remodeling

## Abstract

Much progress in understanding cell migration has been determined by using classic two-dimensional (2D) tissue culture platforms. However, increasingly, it is appreciated that certain properties of cell migration *in vivo* are not represented by strictly 2D assays. There is much interest in creating relevant three-dimensional (3D) culture environments and engineered platforms to better represent features of the extracellular matrix and stromal microenvironment that are not captured in 2D platforms. Important to this goal is a solid understanding of the features of the extracellular matrix—composition, stiffness, topography, and alignment—in different tissues and disease states and the development of means to capture these features

## Introduction

Cell migration is a fundamental process necessary for the creation of tissues during embryogenesis, immune surveillance, wound repair, inflammation, and the invasion and metastasis of cancer cells. All of these processes occur in the context of the extracellular matrix (ECM). For decades, a concerted effort to understand the basic mechanisms of cell migration and other cellular behaviors has focused largely on cells cultured on two-dimensional (2D) surfaces. This body of work has created important knowledge regarding cell migration. Moreover, certain aspects of *in vivo* cell migration are well represented by 2D assays; for example, recapitulating the movement of keratinocytes closing a wound (for example, 1) or capturing melanoma cell migration on deep dermal tissue layers ^2^.

Studies *in vivo* demonstrate that cells also use migratory approaches different from those observed on 2D surfaces *in vitro*, and even 2D migration *in vivo* occurs in the context of 3D tissue. The variety of possible migratory approaches has created controversy about the mechanism of cell migration, the localization of cell signals, and the necessity of cell-matrix adhesions ^3, 4^. Thus, there is a disconnect between what we know about 2D migration and what we might suppose about migration *in vivo*.

Recently, there has been a move to apply the wealth of knowledge regarding what we know about 2D cell migration to understand cell migration within the context of 3D matrices and *in vivo*. Leaders in the field have identified several challenges to this task, which include the fact that migration in 3D matrices *in vivo* is quite different from that in 2D matrices, the difficulty creating relevant *in vitro* matrices that capture matrix composition and topography of *in vivo* microenvironments, and the challenges of manipulating the environment *in vivo* ^5^.

Here, we will investigate features of the ECM that are emerging as key to consider when creating appropriate experimental platforms that can be used to understand the role of the ECM in determining cellular migration, differentiation, development, and pathological processes, and we will discuss ways that these features can be captured by various *in vitro* approaches. Although no single approach can capture all relevant features, by understanding the strengths and limits of different platforms, one can design the most appropriate approach to address specific questions related to cell migration *in vivo*.

## How do we define a relevant matrix?

The ECM can be oversimplified into two major types: interstitial ECM (loose or dense connective tissue) and the basement membrane. The 50–200 nm-thick basement membrane is composed predominantly of laminins, proteoglycans (perlecan and others), nidogen/entactin, and collagen IV. Basement membrane surrounds most epithelial units (ascini and alveoli) and vasculature, providing a defining barrier that provides architectural context and a surface on which epithelial and endothelial cells attach and create basal-to-apical polarity ^6^. Specific differences in basement membranes across different tissues are not well understood.

The interstitial matrix is a complex mixture of proteins that includes predominantly fibrillar collagens supplemented by various proteoglycans and glycoproteins such as fibronectin, laminin, and tenascin. Interstitial matrices vary significantly across different tissues, across developmental time frames, and across disease processes. Interstitial matrices are constructed in an active manner by fibroblasts, which themselves vary across tissues and are altered in pathological conditions. Bone and cartilage are constructed by specialized mesenchymal cells related to fibroblasts: chondrocytes and osteocytes. Other cells such as macrophages, epithelial cells, tumor cells, and adipocytes, are some of the cells that can contribute to ECM production either directly by making ECM proteins or by stimulating fibroblasts to secrete ECM. A complete consideration of how ECM production is regulated has recently been reviewed ^7^.

Thus, depending on the question at hand, the investigator is tasked with determining what is an appropriate ECM in terms of composition and architecture to recapitulate a particular extracellular environment. Mass spectrometry analysis of the ECM had been difficult because of its insolubility, which was overcome recently by important technical advances ^8^. Additional informatics applied to analyzing the proteome of the ECM, termed the “matrisome”, have uncovered important knowledge about the composition of the ECM ^9^. From proteomics, it is clear that carcinoma tissue differs from normal tissue in the composition of the ECM ^10, 11^. From these studies, tenascin C emerges as an important player in mammary carcinogenesis ^12, 13^. Moreover, analysis of metastatic tissue identifies several candidates as possible metastasis promoters ^10, 11^. A complete readout of the matrisome of tissues, such as the lung, allows better tissue engineering approaches ^14^.

## Stiffness and cross-linking

Matrix stiffness has emerged as a key consideration in understanding cellular response to the ECM. Matrix stiffness varies considerably across tissues, and elastic modulus values are around 0.1–1 Pa for neuronal tissue, approximately 8–17 kPa for muscle, and 25–40 kPa for bone ^15– 17^. Moreover, stiffness changes with pathological conditions. For example, whereas normal breast tissue has an elastic modulus on the order of 1.2 kPa, breast tumors are significantly stiffer (moduli of 2.4–4.8 kPa) ^18^. These moduli can be represented by increasing the concentration of collagen gels; gels around 1 mg/mL are similar to normal breast tissue, whereas collagen gels around 4–6 mg/mL have a modulus similar to that of tumors ^18– 20^. The fate of mesenchymal stem cells can be manipulated by matrix stiffness such that a neuronal fate is promoted by culture on a compliant/soft matrix, whereas an osteogenic fate is promoted by a stiff matrix ^21^.

Matrix stiffness increases with the progression of carcinomas and this is due in part to increased deposition of collagen and matrix remodeling ^19, 22^. Moreover, in cancer progression, there is a dramatic upregulation of matrix cross-linkers, including lysyl oxidases (LOXs) and tissue transglutaminases, that correlates to increased matrix stiffness ^23– 25^. The normal physiological role of these cross-linkers is increased stiffness (for example, in dermal wound healing) ^26, 27^. In breast cancer, upregulation of LOX contributes to the increased stiffness surrounding breast tumors ^23^. The importance of accounting for stiffness is demonstrated by findings that altering stiffness dramatically changes the expression of genes and drives a proliferative and metastatic phenotype in breast carcinoma ^19, 22^. Moreover, matrix stiffness alters the response of cells to hormones and growth factors ^21, 23– 28^.

## Imaging the extracellular matrix

It is worth spending a moment discussing how one sees the structure of the ECM, as this has led to experimental questions we might otherwise have failed to ask. The ability to see collagen fibers in live samples without adding exogenous fluorophores is feasible through the techniques of confocal reflectance microscopy, second harmonic generation (SHG), or third harmonic generation (THG). These approaches discern most, if not all, types of fibrillar collagen but cannot discern subtypes of collagen composition. As such fibers represent the major structural feature of the ECM, capturing their structure and organization is often a good place to begin an understanding of what comprises appropriate ECM architecture.

Confocal reflectance microscopy relies on the backward scatter of light and is easily obtained on a standard confocal microscope ^29, 30^, making it readily available to a majority of biologists. Of potential harmonophores, collagen fibrils have a particularly strong SHG signal and are readily imaged ^31– 34^, even in the context of multiple fluorophores (for example ^35^). SHG is the preferred approach for imaging thicker 3D and *in vivo* samples, as the longer wavelengths used can penetrate deeper into tissue, and the use of two photons eliminates out-of-plane focus. SHG misses some fibers, as it depends on the collagen structure to generate the signal. The third harmonic, THG, is useful in combination with SHG, as it captures some structures that are invisible to SHG, including elastin fibers ^2, 33, 36– 38^. Other approaches are being advanced to visualize collagen by using its unique structural properties. Coherent anti-Stokes Raman scattering (CARS) imaging makes use of molecular vibrations to visualize collagen and elastin fibers and discern them from cellular structures ^39, 40^. Optical coherence tomography (OCT) can make use of polarization to discern highly ordered collagen structures such as those in tendon ^41^, and has recently been combined with multiphoton imaging ^42^. There is also interest in exploiting collagen, and the structural information it conveys for meso- and macro-scopic imaging approaches using OCT ^43^, which is being exploited for intra-operative imaging of collagen structures ^44^.

## Topography and alignment

**Figure 1.**
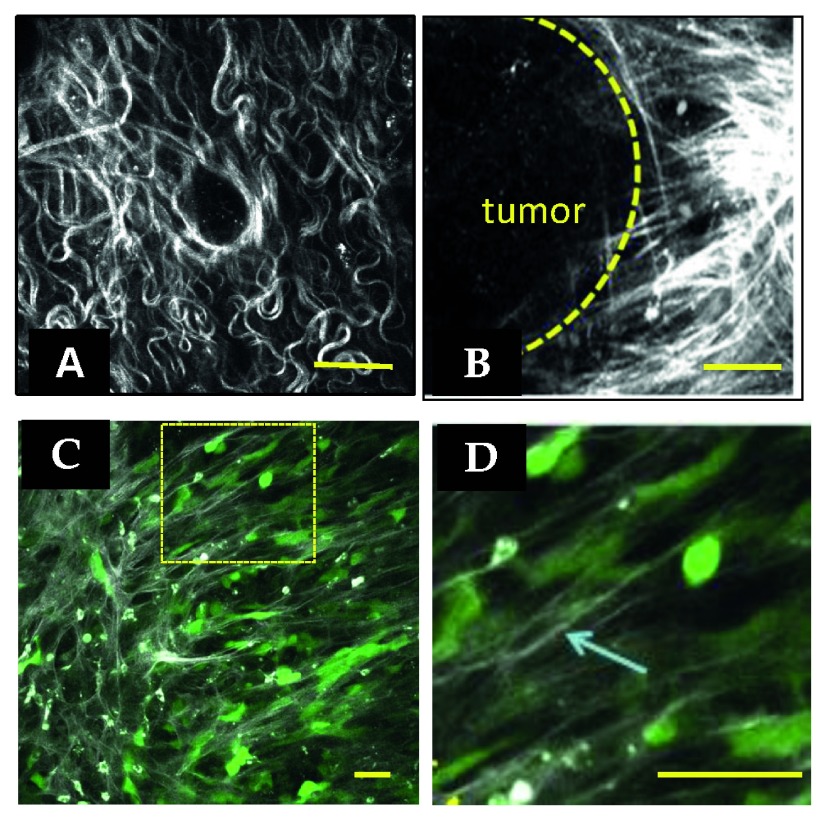
Collagen alignment around tumors facilitates invasion. ( **A**) Second harmonic generation (SHG) image of a normal mouse mammary gland. Collagen appears white. ( **B**) SHG of a mouse mammary PyMT tumor. Note the straightened and aligned collagen perpendicular to the tumor boundary. Both images are reprinted with permission from Provenzano *et al.* ^34^ (2008). ( **C**) Intravital image of human MDA-MB-231 breast carcinoma cells in mouse mammary gland. Cells are transfected with green fluorescent protein. Collagen appears white. Note that cells polarize along collagen. ( **D**) Zoom of boxed region in ( **C**). The arrow points to a single collagen fiber in the field of view. Scale bars = 50 μm.

Using SHG of tissues, the Keely lab has characterized a set of collagen changes, termed Tumor-Associated Collagen Signatures (TACS), that accompany tumor progression ( Figure 1). Notably, these changes manifest in predictable ways and are characterized by the deposition of bundled, straight collagen (TACS-2) that becomes oriented perpendicularly to the tumor-stromal boundary (TACS-3) ^45^. Importantly, these changes are observed in human breast cancer, and the presence of TACS-3 collagen is an independent predictor of poor outcome ^46^. Collagen alignment is also observed in the ECM of the involuting mammary gland during a window in which the ECM demonstrates increased ability to promote mammary carcinogenesis ^47^. Recent findings demonstrate that haplo-insufficiency for collagen III, which can form mixed fibrils with collagen I, leads to an increase in aligned collagen and tumor progression in murine models ^48^. Several additional aspects likely contribute to collagen alignment, as discussed in a recent review ^7^. It is becoming appreciated that the structure of collagen around tumors of various origins in addition to breast carcinoma, including ovarian ^49^, colon ^50^, and prostate ^51^ cancers, changes during cancer progression. It is of interest that the collagen structures of these carcinomas are not identical to each other or to that of breast carcinoma, yet each has a structure that is distinguishable from the normal tissue. A common feature is the increased organization of the collagen to be more aligned, but the actual structure of the collagen (wavy or straight, thick or thin) varies by tissue. Thus, during attempts to capture topography *in vitro*, it will be important to consider tissue-specific structures.

Although the definition of TACS is a useful means to quantify topography in breast cancer, it is becoming clear that a broader means to quantify and describe collagen structure is needed, as there are tissue-specific and disease-specific differences in collagen structure. To address this, a concerted effort has gone into developing image analysis tools to capture and quantify collagen features such as fibril width, alignment, spacing, density, and curliness ( www.loci.wisc.edu/software) ^52^. It is our vision that these additional collagen features will allow the field to provide quantifiers to the structure of the ECM, and to parse out which features may track with pathological changes.

Aligned collagen facilitates the migration of carcinoma cells away from the primary tumor and toward the vasculature ^35, 53– 55^. Cells overwhelmingly track along fibers and are not efficient at migration across the same density of collagen fibers when they are oriented parallel to the cell-matrix boundary ^53^. This observation may reflect the fact that collagen is stiffer along the axis of alignment compared with across the axis of alignment ^56^. Moreover, cells migrating on aligned fibers demonstrate more persistent migration and adopt a bipolar phenotype with fewer lateral protrusions and increased membrane blebbing ^56^, much like the lobopodial migration described by Petrie *et al.* ^3^. As cell migration can be limited by nuclear deformation, especially when proteases are not available, alignment may provide open tracks through which cells move their nuclei ^57^. Although it is intuitively obvious that cells would track along a collagen fiber, the molecular mechanism by which aligned collagen facilitates invasion is unknown. Below, we discuss approaches that should facilitate investigation of migration on aligned fibers.

Matrix remodeling is likely concomitant with cell migration, and the cleavage and straightening of fibers have been observed with careful microscopy ^58^. Moreover, as cancer cells migrate through the ECM, their ability to cleave the matrix can be a distinct advantage ^59^. When protease activity is broadly blocked, cells must change their shape to adapt to the available space because they are more limited by the confinement of the matrix ^57^. One choice cells make to navigate confined spaces is the use of amoeboid migration ^57^. Another choice may be lobopodial migration, which is observed within dense dermal matrices that may be quite confined and is lost when cells are unconfined on top of these same matrices ^3^. Thus, migrating along aligned fibers provides cells with multiple advantages: a stiffer environment on which to move, cues to achieve cellular polarization and limit “distracting” lateral protrusions, and the creation of open areas that allow the nucleus to be readily moved with the cell. The result is efficient and persistent migration on collagen fibers.

## Modeling the complex features of the extracellular matrix *in vitro*

Although animal studies and investigation of cell migration by intravital imaging trump any *in vitro* approach when considering the perfect representation of the relevant ECM, they are limited by the difficulty of the approach. More importantly, it is often difficult to precisely control ECM features in a manner that allows mechanistic understanding of cell response to a particular feature, and this approach is not amenable to large-scale screening or multi-factorial manipulations. Thus, relevant *in vitro* systems are needed to inform and complement the *in vivo* studies.

## Capturing extracellular matrix stiffness

One of the most often overlooked aspects of *in vitro* systems by those not studying mechano- signal transduction is the need to consider ECM stiffness, which profoundly regulates gene expression, stem cell fate, differentiation, and cell phenotype ^16, 19, 21, 22, 28, 60^. Although investigators may not realize it, the majority of *in vitro* experimental approaches set the cellular microenvironment orders of magnitude stiffer than the relevant tissue by coating ECM components on plastic or glass surfaces. In fact, many ignore the ECM altogether by performing experiments on cells cultured on uncoated surfaces, not appreciating that in fact they are culturing on an ill-defined combination of fibronectin, vitronectin, and several other proteins, adsorbing from the bovine serum in the medium onto the plastic. The fact that cell behavior on these surfaces is not the same as *in vivo* cautions us to consider the ways such studies may be limited. For example, in contrast to the more uniformly spread cells that are accompanied by stress fibers and large focal adhesions that are observed on such stiff 2D surfaces, cells in 3D matrices tend to have more elongated shapes, minimal stress fiber formation, and smaller focal adhesions ^3, 61– 64^.

ECM stiffness is most readily captured by the use of polyacrylamide substrata (PAS), in which the amount of bisacrylamide can be varied to tune stiffness in a near-linear manner ^65– 67^. Similar approaches make use of alginate gels or mixed alginate-polyacrylamide ^68^. These surfaces are functionalized by the addition of a cross-linker and then coated with the desired ECM component. The result is the ability to tune stiffness in a precise way and test specific questions about stiffness and cell response. Moreover, nanopatterning allows precise manipulation of the spatial organization and topography of varied stiffness ^66^, and the use of hydrogel columns allows simultaneous measurement of cellular force on the substratum ^69^.

Polyethylene glycol (PEG) hydrogels with controlled stiffness allow incorporation of cells into 3D environments and measurement of forces within ^70^. Hyaluronic acid-based 3D gels demonstrate the importance to stem cell differentiation of adding the third dimension ^71^. Cross-linking of alginate gels with carbonate allows tuning of stiffness ^72^. However, often a limit of hydrogels is that they are dense with minimal pores compared with a natural ECM, and thus cells either confront them as a 2D surface or invade them by uncertain mechanisms. For example, the addition of hyaluronic acid to otherwise soft substrata results in cellular behavior appropriate for stiff surfaces ^73^. In some cases, this property is exploited to create barriers, define geometries, and confinement conditions ^74^.

An additional feature of hyaluronic acid and proteoglycans is their ability to sequester water in the interstitial matrix, which adds to their effect on matrix stiffness. When combined with the effects of pathological conditions such as diabetes, inflammation, cancer, or surgery, all of which can damage or limit lymphatics, the result can be increased interstitial pressure and altered fluid flow. In pancreatic cancer, hyaluronic acid limits adequate perfusion of the tissue with anti-cancer chemotherapeutics, which can be reversed with hyaluronidase ^75, 76^. Interstitial pressure and fluid dynamics are emerging as key regulators of cell behavior, and can enhance cell migration ^77^.

Perhaps the simplest manner in which to create a relevant 3D matrix is the use of collagen gels made from neutralized rat tail or bovine skin collagen. Depending on the extraction method, rat tail collagen typically retains the non-collagenous N- and C-terminal telopeptide domains that allow cross-links of lysine residues. In contrast, collagen obtained from dermis is usually extracted with trypsin, removing the bulk of these regions. These two sources have been directly compared and demonstrate differences in the phenotype and migration of embedded cells ^57^. Stiffness is easily manipulated by contrasting gels that are attached to the culture dish (“restrained” or stiff) compared with those that are released from the dish to float in the medium (“contractible” or compliant; reviewed in 78). Varying the concentration of collagen results in exponential stiffening of the gels over a concentration range of 1–5 mg/mL collagen ^22, 79^.

Various additional ECM components, such as collagen V, elastin, fibronectin, and other proteins, can be added to these gels ^80^. The cross-linking and stiffness of collagen gels can be further modified by the addition of glutaraldehyde, 1-ethyl-3(3-dimethylaminopropyl) carbodiimide (EDC), N-hydroxysuccinimide (NHS) ^81– 83^, hydroxyapatite ^84, 85^, or sugars such as ribose or glucose to cause glycation ^86, 87^. Gels composed of fibrin are also used for several applications; for example, fibrin gels allow endothelial cells to undergo vasculogenesis ^88^. Although gels composed of recombinant basement membrane (matrigel) mimic the composition of the basement membrane, they are not readily manipulated and are too soft to present cells with a stiff environment.

## Capturing extracellular matrix topography and porosity

As described above, the architecture and topography of the ECM have profound ability to alter cellular response. Intravital imaging of carcinoma cell migration on thick collagen fibers (approximately 1–3 μm in thickness) suggests that for some studies it is important to capture this feature ^4, 89^. Recent approaches to recapitulate this sort of migration in simple culture systems have led to the approach of painting thin isolated strips of collagen on a surface to create “1D” cell migration studies ^90^. With this approach, it was recently noted that the spatial organization of the well-known favorite regulators of cell migration, Cdc42, Rac, and Rho, is completely different from when cells are migrating on a 2D surface or within a disorganized 3D collagen matrix ^3, 91^.

Inherently, 1D patterning lacks control of two features that are likely to be important: the diameter of ECM fibers and their stiffness. Cell interaction on fibrous ECM can be categorized at two scales: (a) cells stretching across and interacting with the whole collagen fiber network, and thus responding to bulk material properties or (b) cells interacting with a small number of fibrils or bundles of fibers. Therefore, *in vitro* models mimicking the ECM need to account for both the elastic modulus of the whole mesh, as well as the bending stiffness of individual ECM fibrils of varying diameters. *In vivo*, the tissue architecture varies considerably on the basis of tissue and disease state, and optimal fiber diameter and network pore size result in efficient migration (speed, distance travelled, and persistence). Either a more or a less dense network can lead to less efficient migration: in a dense network, a large number of contacts cause cells to encounter confinement, whereas less dense matrices with larger pores can lead to insufficient contact points for efficient migration ^91– 93^.

**Figure 2.**
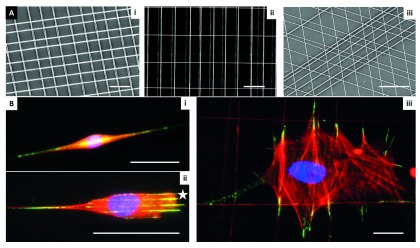
Use of fibronectin-coated fibers on a suspended platform. The platform was created by using the spinneret-based tunable engineered parameter (STEP) technique as described by Nain *et al.* ^100^ (2009). ( **A**) STEP manufacturing platform and scanning electron microscope images of fibers fabricated (i) same diameter crosshatch, (ii) mix of diameters in three layers, and (iii) varying orientations. ( **B**) Immunofluorescence images of single cells in (i) spindle, (ii) parallel with star showing leading edge, and (iii) polygonal shapes (red: f-actin stress fibers, blue: nucleus, and green: paxillin focal adhesion clusters). Scale bars: 10 µm (Ai and Aii), 20 µm (Aiii), 50 µm ( **B**).

One means to precisely control fiber properties is the use of fibers of polycarbonate, or polycaprolactone (polyurethane), which can be combined with collagen ^94, 95^. Arguably, electrospinning is the most widely known and thoroughly studied method of forming polymeric nanofibers. In this process, the polymer solution is pumped through a syringe to a needle where an electrical charge extrudes polymer fibers onto a collecting target ^96, 97^. The principles underlying the process were first observed over 100 years ago, and modern refinements allow electrospinning to generate micro/nanoscale fibers ^96, 98^. With the realization that electrospinning could produce fibers with diameters on the order of those in native tissue, there has been rapid growth in the use and improvement of electrospinning techniques to achieve greater control of alignment and spatial organization. However, the manufacturing challenges in controlling diameter, spacing, and alignment restrict the questions that can be investigated by using electrospinning methods ^96, 98^. Non-electrospinning spinneret-based tunable engineered parameter (STEP) technique is a pseudo-dry spinning nanofiber fabrication technique that does not rely on an electric field to stretch the solution filament, thus allowing arrays of highly aligned fibers to be created. The STEP fiber manufacturing platform allows suspended fibers of a variety of polymers to be deposited with control of fiber dimensions (diameter of less than 50 nm to microns and length in centimeters) and orientation (0–90° and sub-micrometer fiber-spacing in single and multiple layers). With this approach, a network of suspended fibers can be generated that allows the manipulation of fiber stiffness, diameter, and topographical features such as fiber spacing, orientation relative to one another, and the ability to juxtapose fibers of different dimensions, such as micro- and nanofibers ( Figure 2) ^99– 102^. Cells on suspended fibers adapt to the underlying fibrous arrangement, acquiring a spindle morphology on single or parallel fibers, and a polygonal morphology on intersecting fibers.

Fiber arrangements can thus be created to provide cells with simultaneous 1, 2, and 3D cues, by which cells can align along the fiber axis (1D), spread and stretch between fibers (2D), and wrap around fibers, thus sensing the curvature (3D). Indeed, considering the diameter or curvature of the fibers is important, as resulting cell phenotypes are profoundly affected by the diameter of nano- and micro-fibers ^95, 103– 105^. For example, Meehan and Nain show that spindle cell migration speed increases with larger-diameter fibers of similar structural stiffness, but that focal adhesion length decreases ^105^. Cells attached to small-diameter fibers (less than approximately 250 nm) exhibit rounded morphology with active protrusive rates ( Supplementary movie 1), and single cells attached to multiple small-diameter fibers are able to spread ( Supplementary movie 2). This suggests the role of curvature and attachment sites in cell behavior, probably reflecting the need to achieve a minimum threshold area required to establish mature focal adhesions for spreading. Furthermore, through the ability to juxtapose fibers of different dimensions, such as micro- and nanofibers, deposited at different spacing, different migratory modes and associated cell forces can be determined ( Supplementary movie 3 and Supplementary movie 4).

A key feature of migration on a collagen fiber or a 1D strip is that the cell is confined by adhesive choices. Physical confinement due to limited spacing within the ECM is another important feature of the microenvironment that cells must navigate. Various approaches have been used to model this question. The use of microfluidic chambers with widths that vary from 3–50 μm demonstrates that cells are significantly confined and slowed in their migration speed below their nuclear diameter (approximately 10 μm in this study) ^106, 107^. By varying the porosity of collagen gels, Wolf *et al.* determined that cells deform their nucleus during migration but that this is limited; cells cannot traverse through pores smaller than 10% of their nuclear diameter ^57^. The use of proteases to cleave the ECM allows cells to overcome this confinement. Similar results are obtained when a laser is used to etch tracks into 3D collagen matrices, in which there is a lower size limit to tracks through which cells can traverse. Moreover, the tracks guide cells as the path of least resistance ^108^. 3D microchannels have also been used to create physical matrix confinement at defined 2D interfaces. With polydimethylsiloxane (PDMS) pillars to hold up a coverslip under pressure, cells can be forced to migrate through confined spaces, demonstrating the effect on nuclear architecture and profound changes in gene expression ^109^. PDMS microchannels have also been used to create confined spaces through which cells migrate in response to chemotactic gradients ^107^.

An alternative way to engineer topography is through light-based nanofabrication. Here, photoactivation of a hydrogel environment is controlled at the nanoscale to allow incorporation of desired ECM components into a 3D environment ^110^, and has recently been adapted to take advantage of multiphoton microscopy ^111^. Gradients of ECM molecules, including fibronectin, can be created with precise spatial control ^112^. Moreover, the composition can be matched to *in vivo* analysis with spatial control based on imaging; for example, the dense, wavy, and aligned nature of the ovarian carcinoma microenvironment can be recapitulated in terms of nanotopography ^113, 114^. Photochemistry production of defined matrices has been used to control stem cell differentiation and mimic developing heart tissue ^114, 115^. A converse approach is to create a collagen gel scaffold and use multiphoton microscopy to generate microtracks within the scaffold. This approach was used to demonstrate the role of confinement and nuclear deformability as a limiting factor in cell migration ^57, 108^.

## Capturing collagen alignment

A limit to many of the above approaches is that, although they can incorporate collagen, they do not fully capture the collagen fibril or fiber structure that is observed *in vivo.* Collagen assembles into a wide variety of fiber structures on the basis of collagen packing, the incorporation of different collagen types, cross-linking, and the addition of other molecules such as fibronectin or proteoglycans. Although *in vitro* collagen can self-assembly, fibronectin and minor collagens in particular are crucial for collagen fibril assembly *in vivo* (reviewed in 116). Moreover, as detailed above, cells track along collagen fibers by using mechanisms that differ from migration within disorganized matrices ^3, 56^. Thus, there is a great need to create matrices that capture both collagen fiber structure, as well as topography and alignment.

### Engineered collagen matrices

Many approaches take advantage of strain-induced alignment. We have been able to align large gels by strain and then cut out smaller regions to test alignment in the parallel and perpendicular orientations ^56^. Cells themselves can exert strain on the collagen, and a highly aligned region can be created between two plugs of dense cells within a 3D collagen gel ^53^. Force based on shear and flow has also been used to align collagen. Collagen extruded from a syringe into a narrow tube creates a high level of alignment and can be further stiffened with cross-linkers to mimic the stiffness of tissue, such as tendon ^117, 118^.

**Figure 3.**
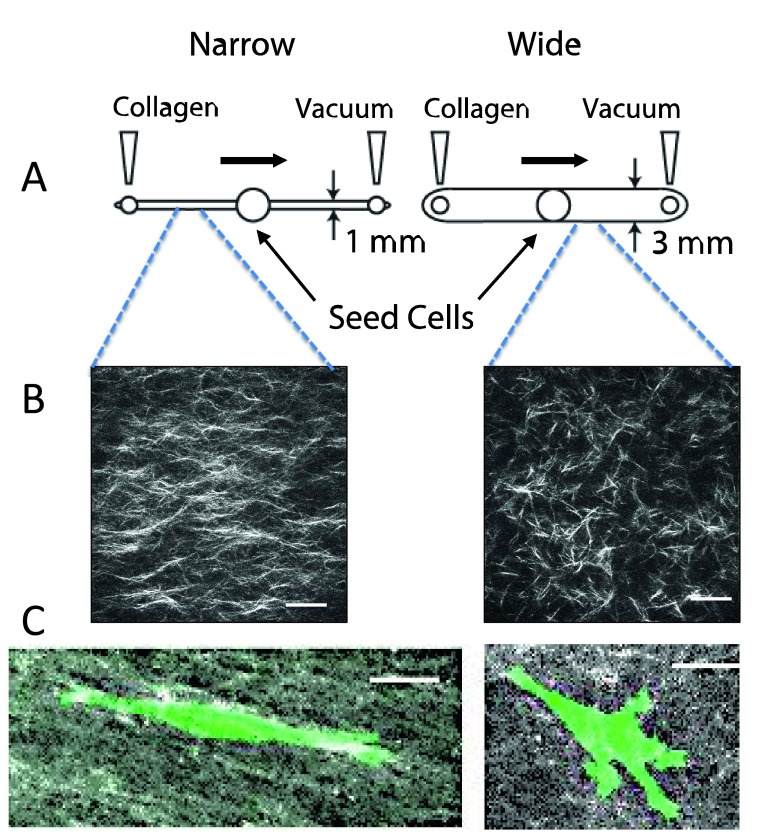
Creation of aligned and random collagen gels in microfluidic channels. ( **A**) Design of microchannels. Collagen is neutralized and pre-incubated for several minutes prior to pulling it across a narrow (1 mm wide × 250 μm tall) or wide (3 mm wide × 250 μm tall) microfluidic chamber. The chambers are incubated for several hours at 37°C, and then the mask for the central port is removed, allowing cells to be seeded into the central port. Cells are incubated and imaged by time-lapse microscopy for 3–4 days. ( **B**) Second harmonic generation image of collagen in a narrow and wide chamber demonstrates aligned fibers in the narrow chamber. ( **C**) Cells in aligned matrices (narrow channel) exhibit elongated morphology and minimal protrusions, whereas cells in random matrices (wide channel) have more abundant protrusions. MDA-MB-231 breast carcinoma cells were transfected with LifeAct-GFP. All images are reprinted with permission from Riching *et al.* ^56^.

We have exploited micro-chambers to generate aligned collagen by using vacuum-induced flow ( Figure 3, 56). If the diameter of the microchannel is narrow (1 mm), an aligned matrix is formed ^68^. These channels can be compared to wider channels (3 mm) in which collagen is randomly organized ^56^. A consideration when working with collagen is that the fibril and fiber diameter can be manipulated by changing the temperature of nucleation and polymerization or by changing the pH of the medium ^57^. Nucleation conditions, polymerization conditions, and flow conditions can be combined to further tune the effects.

### Cell-derived matrices

For many investigations, a single ECM component is used as a substratum for investigation, which is an advantage when the biological question is to compare isolated ECM molecules. However, often the desire is to create a relevant context in which to ask other questions. Recent advances in proteomics demonstrate the molecular complexity of the endogenous ECM ^9^. Even once we know all the components that make up a particular tissue-specific ECM, it may be impossible to re-create the ECM environment *in vitro.* One approach is to culture tissue-specific fibroblasts and allow the fibroblasts to assemble a complex cell-derived matrix (CDM). After several days, the fibroblasts are removed by a gentle alkaline lysis, resulting in a matrix that is 3D at the cellular scale. A nuanced approach is to use tissue-specific mesenchymal stem cells derived from periods of tissue development rather than from the adult. For example, the use of fetal synovium-derived stem cells (SDSCs) creates an ECM that is superior to that of adult SDSCs in promoting chondrocyte differentiation ^119^.

The use of CDMs has allowed an increased understanding of cell adhesions and behavior. By combining this approach with genetically engineered fibroblasts, one can further manipulate the CDM and in many cases achieve a complex matrix that is also aligned ^7^. Overexpression of integrin-linked kinase (ILK) in cardiac fibroblasts leads to increased collagen deposition and fibrosis ^120^, whereas overexpression of fibroblast activation protein (FAP) in fibroblasts leads to deposition and organization of aligned collagen and fibronectin in pancreatic carcinoma ^121^. Deposition of an aligned matrix also requires syndecan-1 expression in fibroblasts ^122^. Conversely, knockdown of caveolin-1 (Cav-1) in fibroblasts results in deposition of a less organized matrix ^123^. By combining fibroblast CDMs with substrata of various stiffness, one can additionally control the microenvironment. In addition, stiffness of the ECM can be achieved by transfection of fibroblasts with LOX ^23, 122, 123^.

A limit of CDM is that generic fibroblast cell lines such as 3T3 cells may not accurately represent the specific ECM of the tissue under investigation and this can be mitigated by using tissue-specific primary fibroblasts or cell lines derived from them when possible. An additional complication is that fibroblasts in healthy adult tissue are not functionally the same as those found in fetal tissues or wound healing, or those associated with carcinomas ^124^. Recent developments using patient-specific fibroblasts to create CDMs show great promise in capturing ECM features of the tumor microenvironment ^125^.

### Decellularized tissues

An alternative approach has been to decellularize isolated tissue and use the remaining ECM as a scaffold for cell studies and tissue engineering. The reader is referred to a recent review that covers several examples of decellularized dermis and other tissues ^126^. Cardiac progenitor cells will migrate into decellularized pericardium and differentiate ^127, 128^. Human decellularized adipose tissue not only promotes the culture of adipocytes but works as an appropriate matrix for breast cells, which exist in the mammary fat pad ^129^. Neuronal matrix scaffolds promote the migration of neural crest-derived cells ^130^, suggesting that there may be usefulness for nerve regeneration. Dermal matrix promotes the migration of keratinocytes to aid healing of wounds and burns ^131, 132^, and tendon matrix promotes stem cell migration and differentiation to repair tendon ^133^. One issue has been the rapid degradation of these matrices *in vivo*, and the addition of PEG into the ECM scaffold adds stability ^134^.

## Adding complexity

As our understanding of cellular behavior deepens, there is an increased need to create complex mimetics of tissue structure. For example, hydrogel-based microfluidic molds can be patterned in a manner that a lumen-based structure is created and surrounded by collagen and stromal cells. In this manner, it is possible to capture features of endothelial-lined blood vessels or epithelial-lined ducts. By the addition of stromal cells, the complex interaction between the endothelium and the surrounding stroma is captured ^135, 136^. Moreover, it is possible to place a breast ductal structure near a blood vessel structure with stroma in between to probe the processes of invasion, intravasation, and extravasation ^137^. Layered microchannel scaffolds of collagen and alginate have been created to mimic the layers of smooth muscle cells that surround blood vessels ^138^. From these approaches, it is now possible to build complexity by including multiple cell types such as macrophages, endothelium, epithelium, smooth muscle, and fibroblasts in the same culture system.

## Summary

With increased understanding of the complex composition and structure of the ECM and how that varies in different developmental stages and during normal and disease processes, it will be possible to create *in vitro* microenvironments that better capture the complex *in vivo* ECM. Such relevant ECMs will allow a more complete understanding of basic questions related to cell migration, wound healing, and a variety of other cellular behaviors. By adding tissue-specific cells, fibroblasts, vascular components, and immune cells, the bi-directional signaling between these compartments will be more readily investigated. In addition to advancing our understanding, these approaches should lead to the development of disease-specific and personalized tissue mimetics for testing drug efficacy across a variety of patients and conditions.

## Supplementary materials

**Movie 1. Cells attached to small-diameter fibers.** STEP (spinneret-based tunable engineered parameter)-based fiber assembly of two small-diameter fibers (less than approximately 250 nm) suspended between two large-diameter fibers (north-south direction, diameter of approximately 2 µm). Cells attached to small-diameter fibers (approximate location shown by two arrows) exhibit rounded morphology, and active protrusive rates suggest insufficient surface area to mature focal adhesions. Cells attached to the large-diameter fibers are stretched in spindle morphologies. Time is presented in hours:minutes:seconds

**Movie 2. Cell attached to multiple small-diameter fibers.** STEP (spinneret-based tunable engineered parameter)-based fiber assembly of multiple small-diameter fibers (less than approximately 250 nm) suspended between three large-diameter fibers (north-south direction, diameter of approximately 2 µm). Single cell attached to multiple small-diameter fibers is able to stretch and migrate while simultaneously putting multiple protrusions on individual fibers (shown by arrows). Time is presented in hours:minutes:seconds

**Movie 3. Cells on single and parallel fibers.** STEP (spinneret-based tunable engineered parameter)-based fiber assembly of aligned fibers with varied spacing causing cells to form spindle and parallel shapes of varying widths, each having different migratory response. Time is presented in hours:minutes

**Movie 4. Cells on single and intersecting fibers.** STEP (spinneret-based tunable engineered parameter)-based fiber assembly of aligned parallel and intersecting fibers. Cells attached to single fibers form spindle morphologies, while a dividing cell at the intersecting fiber forms polygonal shape (kite) and then emerges on the single fiber as spindle. Time is presented in hours:minutes
